# Development and Early Implementation of a Public Communication Campaign to Help Adults to Support Children and Adolescents to Cope With Coronavirus-Related Emotions: A Community Case Study

**DOI:** 10.3389/fpsyg.2020.02184

**Published:** 2020-09-10

**Authors:** Daniela Raccanello, Giada Vicentini, Emmanuela Rocca, Veronica Barnaba, Rob Hall, Roberto Burro

**Affiliations:** ^1^ Department of Human Sciences, University of Verona, Verona, Italy; ^2^ Environmetrics Pty Ltd., Killara, NSW, Australia

**Keywords:** coronavirus, emotions, coping strategies, communication campaign, children, adolescents

## Abstract

Epidemics and pandemics can traumatically impact the emotional wellbeing of adults, children, and adolescents in diverse ways. This impact can be reduced by applying a range of evidence-based coping strategies. Based on previous research, we created a pamphlet-based communication campaign designed to assist adults to provide support for young people confronted with emotional distress associated with the pandemic caused by the novel coronavirus [severe acute respiratory syndrome coronavirus 2 (SARS-CoV-2)] and the related disease [coronavirus disease (COVID-19)] in 2020. We developed a pamphlet describing the common emotions children and adolescents report feeling in the face of disasters and the coping strategies that have proven effective in mitigating them. The target population was adults who interact with children and adolescents in both formal and informal settings. The pamphlet included basic information on this specific emergency, emotions that might be commonly experienced, and coping strategies for dealing with negative emotions. The aim of this paper is to describe the planning, development, and implementation of the campaign. First, we monitored how the media gave visibility to the campaign during the 40 days following the release of the pamphlet: it potentially reached a large audience at a national and international level through at least 216 media channels included the HEMOT^®^ (Helmet for EMOTions) website. Second, Google Analytics™ data from the HEMOT^®^ website enabled us to examine the characteristics of the visitors to the website and the behavior of those who viewed the pamphlet. More than 6,000 visitors, most from Europe followed by the Americas, visited the website in the first 40 days after the pamphlet publication. The webpage including the pamphlet obtained over 6,200 views, most directly or *via* other websites. A cluster analysis suggested that the access to the webpage did not mirror the trend concerning the new cases of COVID-19 in Italy (which increased during the central phase of the campaign) or worldwide (which continued to increase across the 40 days). Third, data gathered with a convenience sample of adults who had consulted the pamphlet provided a perspective on the comprehensibility of the messages conveyed by the pamphlet and on the utility for children and adolescents. The process we have demonstrated in this example could be replicated in different communities and settings to respond to the spread of the COVID-19 or to respond to other widespread or more localized disasters.

## Introduction

Epidemics and pandemics constitute public health problems that can have a highly traumatic impact on people’s psychological functioning (Ko et al., 2006; Goodwin et al., 2011; Prati et al., 2011; Vaughan, 2011; Galea et al., 2020; Kwok et al., 2020; Shigemura et al., 2020; Xiang et al., 2020). This is especially true for children and adolescents, whose vulnerability depends on their level of cognitive and emotional development (Kar, 2009; Bouffet et al., 2020). To our knowledge, no studies have yet addressed their emotional reactions during epidemics and/or pandemics. Health professionals have a range of evidence-based techniques for teaching young people strategies to overcome negative feeling (Flay et al., 2005; Gottfredson et al., 2018). Nevertheless, if emergencies occur, lay adults need rapid access to simple tools to assist young people cope with the situation (Galea et al., 2020; Horesh and Brown, 2020; Xiang et al., 2020).

Therefore, we developed a communication campaign to help adults support children and adolescents cope with negative emotions during a public health emergency of international concern (PHEIC) and, in particular, the pandemic related to the spreading of the novel coronavirus [severe acute respiratory syndrome coronavirus 2 (SARS-CoV-2)] and the related disease [coronavirus disease (COVID-19)] in 2020. The campaign was promoted within the HEMOT^®^ project (Helmet for EMOTions), a larger project focused on emotional preparedness related to disasters. In the context of quite an amount of misinformation being circulated, it was important to develop reliable and authoritative sources relying on scientific literature (Bouffet et al., 2020).

## Background and Rationale

### Psychological Consequences of Epidemics/Pandemics

Epidemics and pandemics can be classified as biological natural disasters (EM-DAT, 2020). Traumatic consequences of natural disasters include impaired health (e.g., cardiovascular ailments), psychopathology (e.g., posttraumatic stress disorder and depression), and negative emotional impact (e.g., anxiety, fear, and anger), both for primary victims experiencing the events directly and for secondary victims indirectly affected through media exposure (Galambos, 2005; Neria et al., 2008; Furr et al., 2010; Masten and Osofsky, 2010; Fergusson and Boden, 2014; Galea et al., 2020). In the case of COVID-19, media coverage can amplify secondary traumatization (Garfin et al., 2020). Psychopathologic symptoms such as depression have been documented for pandemics such as the severe acute respiratory syndrome (SARS; Ko et al., 2006) and in the case of COVID-19 (Wang et al., 2020). One of the few studies focusing on the psychosocial consequences of COVID-19 reported around 6% of Chinese adults experiencing anxiety and 17% experiencing depression during February 2020. This underlines the need for mental health support in such situations (Wang et al., 2020). However, for most disasters, the traumatic consequences are a result of the disaster itself, while for COVID-19 the traumatic impact is amplified by the measures, such as social distancing, used to limit the spreading of the virus (Galea et al., 2020).

Other studies described the emotional impact of different infectious diseases. Fear and anxiety are the most salient emotions during an influenza outbreak, followed by anger and sadness (Kim and Niederdeppe, 2013). Studies on infectious diseases such as the swine flu pandemic, the avian influenza, and the SARS also show that fear, regret, and worry are associated with attempts to keep free from the disease along with managing the disruptions to normal life (Goodwin et al., 2011; Prati et al., 2011; Vaughan, 2011; Karademas et al., 2012; Manabe et al., 2012). However, excessive levels of fear can transform into panic and have serious detrimental effects, like the so-called “SARS phobia” (Cheng and Tang, 2004).

To our knowledge, little specific data on how the COVID-19 has affected people’s emotional reactions and the ways they cope with them have been published (Kwok et al., 2020; Wang et al., 2020). The salience of emotions such as fear has been evidenced by the rapid creation of instruments like the Fear of COVID-19 Scale (Ahorsu et al., 2020). Fear, with distorted perceptions of risk, could contribute to negative societal behaviors and serious public mental health concerns linked to COVID-19 (Shigemura et al., 2020).

### Coping and Epidemics/Pandemics

A psychological process helping to diminish the traumatic impact of a disaster is “coping,” a multi-component construct referring to all the actions marshaled to face stressful events (Skinner et al., 2003). During and after disasters, children can use a large variety of coping strategies to feel better. A meta-analysis (Raccanello et al., 2020a) examined the relation between coping strategies used after a disaster and indicators of persistent traumatic symptoms or positive changes over time among children and adolescents. In that study, we coded coping strategies into three categories (Table 1) according to the developmental classification of Zimmer-Gembeck and Skinner (2011), with each category corresponding to different adaptive functions, and including two related strategies and their opposites. One set, termed “problem-focused strategies”, involves problem-solving and information-seeking/giving in contrast to feeling helpless and seeking escape. This approach helps individuals to adapt their behaviors to the environmental constraints they face. The second set was called “relation-focused strategies” and it involves self-reliance and support-seeking/giving in contrast to delegation and social isolation. These revolve around endeavors to build reliance among and between people caught up in the disaster. The third category, “priority-focused strategies”, features accommodation and negotiation in contrast with submission and opposition. These actions are organized around efforts to “trade” options to reach one’s own goals. This meta-analysis has confirmed the expected efficacy of these strategies in mitigating the negative effects of disasters, and its results were further confirmed relating earthquakes (Raccanello et al., 2020a). It showed that strategies incorporating escape, delegation, social isolation, and opposition were positively linked with traumatic symptoms, while problem-solving and support-seeking actions were positively linked with indicators of positive change (e.g., self-efficacy and understanding of emotions). Submission was ambiguously related to both negative and positive indicators.

**Table 1:** Functions of coping strategies (Zimmer-Gembeck and Skinner, 2011), labels used in the pamphlet, and description.

Adaptive function	Coping strategies	Label used in the pamphlet	Description
Problem-focused strategies	Problem-solving	Try to solve the problem	Concentrating on the problem, aiming at changing the situation to find a solution
	Information-seeking/giving	Talk about facts	Searching for information and giving information
	Helplessness	Give up	Giving up, being passive or confused in front of the requests
	Escape	Ignore reality	Avoiding the problem, through behaviors or cognitions
Relation-focused strategies	Self-reliance	Understand and express your emotions	Counting on oneself, through emotional expression and regulation
	Support-seeking/giving	Receive and give help	Seeking/giving social, concrete, emotional, and/or instrumental support
	Delegation	Put the responsibility on to others	Assigning the responsibility of the solution to others, complaining or self-pitying
	Social isolation	Isolate yourself from others	Disengaging from or refusing social interactions
Priority-focused strategies	Accommodation	Take some time to focus on other things	Adapting smoothly to alternatives and focusing on positive aspects
	Negotiation	Adapt	Seeking new alternatives, such as finding compromises and allocating priorities
	Submission	Continue to think negatively	Giving up, ruminating, or having a rigid attitude
	Opposition	Ignore the recommended safety plans	Rejecting collaboration or doing the contrary as regards requests

These data are in line with studies involving adults in pandemics such as the swine flu, indicating that anxiety is negatively related to problem-focused strategies (i.e., problem-solving, cognitive restructuring, social support-seeking, active distraction, and humor) and positively to emotion-focused strategies (i.e., self-blame, other blame, rumination, wishful thinking, emotional containment, emotional expression, cognitive distraction, and passive resignation; Taha et al., 2014). However, another study focused on swine flu indicated, ambiguously, that both negative and positive emotions were associated with strategies such as information-seeking and that positive emotions were related to relational trust (Kim and Niederdeppe, 2013). A further study indicated that using different coping strategies buffered the negative influence of SARS-related stressful events on perceived general health, but that using avoidant strategies was positively related to developing psychological symptoms such as somatization, obsessive-compulsive, depressive, and anxiety symptoms (Main et al., 2011). These previous studies can be linked to the observation that the ability to recognize, understand, and regulate one’s emotions offers a potential protection factor against the traumatic impact of disasters and, in particular, of pandemics (Denham, 1998).

### The Present Study

In line with this literature, we identified the contents of the campaign using a preliminary model of emotional preparedness for disasters that incorporates developmental changes, developed within the PrEmT project, “Emotional Prevention and Earthquakes with primary school children” (Raccanello et al., 2019, 2020b,c). The model is supported by data derived from an evidence-based intervention which involved primary school children. The data revealed an increase in children’s semantic knowledge regarding earthquake-related coping strategies for those children who participated to the intervention compared to the participants belonging to a control group. The model takes into account the interactions between the perception of sensory stimuli, working memory elaboration, and enactment of behaviors. In particular, it describes how affect can impair the psychological functioning during an earthquake, on the basis of the literature which supports the bi-directional relations between emotions and perception, memory, and enactment of behaviors. It focuses on possible emotions that could be felt during and just after an emergency situation and on coping strategies useful to manage them. The key relevance of this model for disaster preparedness concerns the relation between the encoded semantic knowledge and its retrieval when an emergency occurs. If relevant knowledge on how to behave safely and cope with one’s emotions has been incorporated in advance of an emergency, receiving a message based on validated persuasion models is likely to increase one’s probability of becoming resilient in the face of adversities.

The aim of this paper is to describe the planning, development, and implementation of a mechanism for providing psychological tips for dealing with the emotions young people might feel in response to the coronavirus pandemic. A pamphlet was designed to provide direction for adults who needed to give emotional support to children and adolescents during the PHEIC and the pandemic triggered by the SARS-CoV-2 and the related COVID-19 in 2020. It was initially targeted to Italian and English speakers, but it was then extended to a variety of other languages. In the literature, previous findings on pamphlets’ efficacy as a psycho-educational resource are lacking, with some exceptions. Most of them are focused on the efficacy of pamphlets addressed to adults. For example, Shah et al. (2018) tested a pamphlet on health and mood addressing people older than 55 years, in order to increase their will to be screened for depression. Garcia et al. (2010) developed an educational pamphlet to increase knowledge on oncological children’s nutrition addressed to low-literacy caregivers. Other researchers found that a pamphlet on proper antibiotic use was more efficacious for increasing knowledge of parents who consulted it compared to a control group who did not consult any material, although it was less efficacious than an animated video (Schnellinger et al., 2010). Interestingly, some authors (King et al., 2003) documented that children and adolescents’ readability of pamphlets concerning mental health is satisfactory when it is characterized by: signaling devices (such as titles, subtitles, and introductory statements) and pronoun references, substitutions, and connectives, to guarantee global and local coherence; unity, such as focus on a single topic; audience appropriateness; definition of technical words; incorporation of questions; and attention to specific attributes of typography variables such as font size, type of print, and color.

We monitored the campaign during the 40 days following the release of the pamphlet, from the 28th February to the 7th April 2020. First, we traced how the media gave visibility to the campaign (aim 1). Second, we used Google Analytics™ data from the HEMOT^®^ website, from which the pamphlet could be downloaded, to assess the characteristics of the visitors and their behavior, also relating the number of views with the number of new cases of COVID-19 in Italy and worldwide (aim 2). Third, about one week after the 40-day period, we recruited a convenience sample to evaluate the perceived comprehensibility and utility of the pamphlet (aim 3).

## Description of the Case

The novel coronavirus is a virus identified for the first time in China on 9th January 2020 (EpiCentro, 2020b; World Health Organization, 2020b). Its official name is SARS-CoV-2, while the name of the related disease is COVID-19, as announced on 11th February 2020, respectively, by the International Committee on Taxonomy of Viruses (ICTV; World Health Organization, 2020a) and by the World Health Organization, WHO. The diffusion of COVID-19 led the WHO to declare a PHEIC on 30th January 2020 (World Health Organization, 2020d). A PHEIC is “an extraordinary event determined to constitute a public health risk to other states through the international spread of disease and to potentially require a coordinated international response” (World Health Organization, 2016, p. 9). On 11th March 2020, the WHO declared a pandemic, an even more extraordinary event which “occurs when a new influenza virus emerges and spreads around the world, and most people do not have immunity” (World Health Organization, 2020c).

### Setting

In Italy, the first two cases of COVID-19 were certified on 30th January 2020 (EpiCentro, 2020a). Since then, a sequence of ordinances and decrees (Presidenza del Consiglio dei Ministri, 2020) introduced measures to limit the spreading of the virus, resulting in severe constraints on freedom of people including increases in social distancing. Measures pertaining to “lockdown” were extended, involving 11 municipalities in the regions of Lombardia and Veneto (from 23rd February 2020); then the region of Lombardia with 14 provinces in the regions of Veneto, Emilia-Romagna, Piemonte, and Marche (from 8th March 2020); and then the whole Italy (from 9th March 2020). Schools were closed beginning on 24th February 2020. When the process evaluation of this campaign ended, all these measures were still present.

On 28th February (which was the day in which the pamphlet was released), in Italy, a total of 888 people had been reported infected. Of these, 412 were in isolation at home, 409 in hospital (64 of them in intensive care units, ICU), 46 recovered, and 21 had died (Protezione Civile, 2020). On 7th April (which was the last day of monitoring of the campaign), in Italy, a total of 135,586 people had been reported infected. Of these, 61,557 were in isolation at home, 32,510 in hospital (3,792 in ICU), 24,392 recovered, and 17,127 had died (Ministero della Salute, 2020a). Worldwide, on 7th April, there had been 1,214,973 confirmed cases since the beginning of the outbreak, 67,841 deaths, and 208 countries, areas, or territories with cases (Ministero della Salute, 2020b). Children represented less than 5% of the diagnosed cases, and they had milder symptoms compared to adults (Ludvigsson, 2020). We represented in Supplementary Figure 1 the daily number of new cases of COVID-19 in Italy and worldwide as reported in the Dashboard by the Center for Systems Science and Engineering (CSSE) at Johns Hopkins University (Dong et al., 2020; numbers higher than 1,000 are rounded to the nearest 1,000).

### Collaborative Partnerships

The campaign was carried out by a multi-disciplinary team with a strong psychological orientation within the HEMOT^®^ project. The HEMOT^®^ project focuses on emotional preparedness in case of disasters, by conducting research and developing training programs for children, adolescents, and adults.

The multi-disciplinary nature of HEMOT^®^ led to collaborative partnerships with teachers who checked the clarity of the message and with the University of Verona and the Civil Protection of the Veneto Region. The latter was actively involved in managing the emergency caused by the novel coronavirus in the local territory of the Veneto region during 2020. Both organizations authorized the inclusion of their logos in the pamphlet.

## Materials and Methods

### Target Population

The target population consisted of adults who might come into contact with young people during the emergency. It was important to reach this population as quickly and as extensively as possibly because one of the first actions taken to limit the spreading of the virus was the closing of schools, placing on family members and carers the task of explaining the emergency and the related mitigation measures.

In the beginning, the campaign addressed Italian and English speakers. We progressively translated the pamphlet into another 15 languages, i.e., Arabic, Croatian, Finnish, French, German, Greek, Lingala, Moldavian, Norwegian, Portuguese, Romanian, Sinhalese, Spanish, Swahili, and Swedish.

### Campaign Content

We developed the content of the campaign by adapting training on emotional management previously tested with children within the PrEmT project (Raccanello et al., 2019, 2020b,c). First, we identified the message to be conveyed by the pamphlet (**Appendix**). The title of the pamphlet focused people’s attention on the problem, i.e., the *public health emergency*, on possible solutions, i.e., referring to *psychological tips*, and on the final target audience, i.e., *children and adolescents*. The pamphlet was divided into three sections with basic information on the problem, emotions that could be experienced, and coping strategies. The contents of the first section relating to the questions *What is a coronavirus?* and *What is a public health emergency?* included the definitions used by WHO (World Health Organization, 2016, 2020a,c). When the pamphlet was released, WHO had not declared a pandemic yet. As part of the pamphlet design, we included an image representing the novel coronavirus.

The second section introduced by the question *Which emotions can we feel?* included both verbal labels and drawings of faces representing emotions that might be felt during a PHEIC. There was a statement suggesting that people can feel three basic negative emotions, i.e., fear, sadness, and anger, but that it would be *great to continue to feel emotions such as relaxation and enjoyment*. The choice of the emotions was based on the PrEmT training and on the literature on disasters (Raccanello et al., 2019, 2020b,c), specifically on epidemics and pandemics (Goodwin et al., 2011; Prati et al., 2011; Vaughan, 2011; Karademas et al., 2012; Manabe et al., 2012; Kim and Niederdeppe, 2013), and it was confirmed by a pool of six experts in psychology (among whom there were a primary school teacher and three parents). We presented both verbal labels and faces for two reasons. First, the presentation of drawings could facilitate the recognition of the corresponding emotions. Second, this was in line with the media richness theory, according to which combining information presented as text and drawings would facilitate effective communication (Hsieh and Tseng, 2017).

The faces had been drawn *ad hoc* within the PrEmT project and had been tested for validity in a pilot phase (Raccanello et al., 2020c). The pilot involved 233 second and fourth-graders in a naming task, in which we had shown the five faces and asked to produce a label that described each emotion being illustrated. Most children had reported the appropriate label for the basic emotions (fear: 75.11%; sadness: 90.13%; anger: 98.71%; enjoyment: 95.71%), while the percentage of correct responses was somewhat lower for relaxation (59.23%). This is consistent with the fact that calm/relaxation is not a basic emotion for which there is a unique correspondence between facial expression and emotion (Ekman, 1992, 1993). But given the key role of this emotion for emergency-related situations, it was appropriate to include it. For ethical issues, the faces were balanced for gender (male and female) and ethnic origin (European, Asiatic, and African).

The third section of the pamphlet presented tips for responding to the question *How can we cope with fear, sadness, and anger?* We rephrased the names of the 12 coping strategies and their three corresponding functions to increase their comprehensibility in general, but especially for children (Table 1). We used the label *Look for solutions* for problem-focused strategies; *Seek and give support* for relation-focused strategies; and *Understand what is important* for priority-focused strategies. We distinguished strategies considered adaptive in the literature (*Dos*) from strategies usually not adaptive (*Don’ts*), stating in the pamphlet that individual and contextual factors can play a role for influencing their adaptivity (Zimmer-Gembeck and Skinner, 2011). For each category, we inserted two/three examples. Some examples were general and pertained to any disaster (e.g., *Talk about how you feel* among *Dos*; *Panic* among *Don’ts*), while others related specifically to quarantine or social distancing due to the coronavirus emergency (e.g., *Do the right things*, *for example*, *washing your hands frequently* among *Dos*; *Ignore the regulations from the Ministry of Health* among *Don’ts*). We identified the examples deductively adapting items from the HEMOT^®^ web application developed within the PrEmT training and inductively through content analysis by six experts in psychology.

The pamphlet concluded by inviting people to invent new ways to cope with negative emotions. It highlighted that the efficacy of coping strategies can vary according to different cases, i.e., individual characteristics, contexts, and also at different times in the same contexts (Zimmer-Gembeck and Skinner, 2011).

### Campaign Dissemination

The campaign was disseminated through formal and informal communication channels. The formal channels were both internal and external to the University of Verona. On 28th February 2020, we uploaded the pamphlet within the HEMOT^®^ website (see the following section). The press office of the University of Verona published an article on the campaign in the online UnivrMagazine and sent a press release to external media. We, then, contacted other media directly. We also asked the School Office of the Veneto region to disseminate the campaign in the Veneto region. From 23rd March to 1st April 2020, we also disseminated the link from which the pamphlet could be downloaded among students from the University of Verona, within a research. Informal channels included a variety of personal contacts and social networks such as Facebook, Twitter, or LinkedIn and instant-messaging technologies such as WhatsApp, Telegram, or others. We monitored the diffusion of the pamphlet through a search conducted daily using the Google Chrome™ browser.

### Website Monitoring

Within the website HEMOT^®^^1^, we created a dedicated webpage to upload the pamphlet^2^. The HEMOT^®^ website is aimed at disseminating all the activities pertaining to the HEMOT^®^ project. It was created using WordPress, a free and open-source content management system. The menu on the homepage includes these links: Home, Mission, and Latest News. The pamphlet was accessible from the Latest News tab. The website also includes five other pages, i.e., Our Team, About Us, Copyright & Disclaimer, The Project in Brief, and Selected Publications. The website is in English and the corresponding domain was registered during June 2019.

Monitoring the Google Analytics™ data related to the HEMOT^®^ website gave us the opportunity to gather data on how visitors behaved and their location (Kirk et al., 2012; Crutzen et al., 2013). Google Analytics™ data contain no personally identifiable information (Kirk et al., 2012). We examined the following measures.

#### Measures

##### Characteristics of the Visitors of the Website

We assessed: (a) the number of users who had initiated at least one session during the 40 days following the publication of the webpage on coronavirus-related pamphlet (28th February–7th April 2020) and the number of users of the 40 previous days (19th January–27th February 2020); (b) the location of users between 28th February and 7th April 2020.

##### Behavior of the Visitors of the Webpage Containing the Coronavirus-Related Pamphlet

Between 28th February and 7th April 2020, we collected data on: (a) the total of pageviews, including repeated views of a single page, and the average time on page, i.e., the average time users spent viewing the page; (b) the location of the users that viewed the webpage; (c) traffic sources, indicating where visitors came from: direct traffic (i.e., directly to the webpage), referrals (i.e., through links at other websites), social traffic (i.e., through links in social networks), and organic search (i.e., after a search engine query).

### Perceived Comprehensibility and Utility of the Pamphlet

We recruited a convenience sample of 144 adults (*M*_age_ = 32.9 years, *SD* = 13.6; 83% females); 21.5% of them had sons and/or daughters under 18 years old; 27.1% worked with children and/or adolescents (as a teacher, educator, etc.). In conducting this part of the study, we followed the ethical standards of the American Psychological Association, presenting the consent form. Specifically, all the adults gave their written authorization for the participation to the research and for data treatment according to the European regulations (679/2016, art. 13). In addition, the software used for the data gathering guarantees ethical standards and data protection through HTTPS security protocols. HTTPS enables encrypted communication and a secure connection between a remote user and our web server. We recruited the participants involving people to whom we had previously sent the pamphlet (e.g., students from the University of Verona, and teachers and parents to whom we had sent directly the pamphlet). We administered an online survey beginning one week after the 7th April 2020 (it could be completed during the following week). We assessed perceived comprehensibility of the pamphlet by adapting questions reported in the psychological literature (e.g., Atkin and Freimuth, 2001; i.e., *How clear is the message conveyed by the pamphlet?*) and utility for children and/or adolescents (i.e., *How useful is the message conveyed by the pamphlet for children and/or adolescents?*). Responses had to be rated on a five-point scale (1 = *not at all* and 5 = *very much*). Despite possible limitations of single-item measures (e.g., low variance and reduced validity measuring a complex construct), previous studies support their reliability and, thus, their utility in situations where surveys need to be as brief as possible (Goetz et al., 2006).

## Results

### Monitoring of Dissemination Through the Media

The pamphlet was accessed both in Italy and abroad. Between 28th February and 7th April 2020, the pamphlet was advertised by 12 Italian newspapers (e.g., Il Sole 24 Ore, L’Arena, and Il Mattino di Padova) and one American newspaper, i.e., The New York Times. Moreover, it was mentioned within the Italian television program “Primus inter pares” on TV7 Triveneta. The pamphlet was also promoted through a variety of school and university channels. In Italy, the School Office of the Veneto region advertised the campaign through its website devoted to all the head teachers of preschool, primary, and secondary schools in the Veneto region. This led to the uploading of messages relating to the pamphlet in at least 84 school/university websites or Facebook pages in Veneto and other Italian regions. Abroad, reference to the pamphlet appeared in at least 34 school/university websites or Facebook pages, mostly in the United States of America. Other websites (*n* = 22) or social network pages (*n* = 59) managed by a variety of professionals such as psychotherapists, psychologists, doctors, pediatricians, physiotherapists, and institutions such as municipalities and associations referred to the pamphlet, both in Italy and abroad. Finally, the pamphlet was cited in a scientific paper published by *Pediatric Blood & Cancer*, that included it in a list of 17 reliable sources for recommendations for pediatric and oncology agencies regarding COVID-19 (Bouffet et al., 2020). To sum up, the pamphlet was advertised through at least 215 media channels, excluded the HEMOT^®^ website.

### Website Monitoring

#### Indicators From Google Analytics™

We examined the following indicators from Google Analytics™ linked to the HEMOT^®^ website.

##### Characteristics of the Visitors of the Website

During the 40 days following the publication of the webpage with the coronavirus-related pamphlet, 6,090 users visited the website. The number of visitors increased massively compared to the number of users in the previous 40 days (*n* = 44). Between 28th February and 7th April 2020, the location of the majority of website users was Italy (67.60% were Italian, while 32.40% were non-Italian), as expected. But the campaign also had an impact at the international level: 75.29% of the users came from Europe (including Italy); 22.45% from Americas; 1.24% from Asia; 0.68% from Oceania; and 0.01% from Africa. The location of 0.33% of users was not identified.

##### Behavior of the Visitors of the Webpage Hosting the Coronavirus-Related Pamphlet

The total pageviews of the webpage with the pamphlet was 6,236, with an average time on page of 2 min and 58 s. Peaks in the number of users (i.e., higher than 200 users per day) were associated with specific events (Supplementary Figure 1), i.e., the dissemination through The New York Times and the School Office of the Veneto region (2nd–5th March 2020); the first work day after the decree which extended the “lockdown” to Lombardia and other 14 provinces (9th March 2020); the dissemination to the participants in the research on coronavirus-related emotions at the University of Verona (25th, 30th March, and 1st April 2020). As regards the users’ locations during the three waves of views, most users came from Europe (respectively, 96.32, 89.81, and 91.59%), followed by Americas (3.42, 10.19, and 8.09%); during the first and third waves, a lower percentage of users came from Oceania (0.13 and 0.19%) and Asia (0.13 and 0.19%). However, across the whole period, the users came from many parts of the world (Supplementary Figure 2). On the whole, users arrived at that page through four channels. Direct traffic led to 59.49% of the visits in the webpage, referrals to 33.45%, organic search to 4.73%, and social traffic to 2.33%.

#### Daily Pageviews and Daily New Cases

In addition, we examined the relation between the number of daily pageviews of the webpage with the pamphlet from 28th February to 7th April 2020 and the number of daily new cases in Italy and worldwide in the same period (Dong et al., 2020; Supplementary Figure 1). In order to explore these relations, first, we calculated the correlations between the three measures and, second, we conducted a *k*-mean cluster analysis (MacQueen, 1967) and generalized linear models (GLMs) with the R software (R Core Team, 2020).

##### Correlations Between Daily Pageviews and Daily New Cases

There was a significant correlation between the number of daily pageviews and the number of daily new cases in Italy (*r* = −0.39, *p* = 0.012). The correlation was moderate and negative, indicating that, on the whole, the number of daily pageviews decreased while the cases unluckily increased as time passed. Another significant correlation emerged between the number of daily new cases in Italy and worldwide (*r* = 0.71, *p* < 0.001). In this case, the correlation was strong and positive, suggesting that, even if Italy was one of the first European countries to be interested by COVID-19, the trend of its new cases was in line with what was happening worldwide.

##### Cluster Analysis on Daily Pageviews and Daily New Cases

To examine deeper these relations, we ran a *k*-mean cluster analysis. We considered the number of daily pageviews and the number of daily new cases in Italy and worldwide, in order to identify whether the days from 28th February to 7th April 2020 could be grouped into different phases with similar characteristics. Even if the extant literature does not report standard rules for determining the minimum sample size for conducting cluster analyses (Mooi and Sarstedt, 2011), Formann (1984) suggested a minimum of 2*^k^* cases, with *k* as the number of variables included to form the clusters. We used three variables, i.e., the number of daily pageviews and the number of daily new cases in Italy and worldwide. In our case, the minimum sample size resulted eight, indicating that our sample size was adequate. Therefore, we ran the *k*-mean cluster analysis on the 40 days.

**Figure 1:**
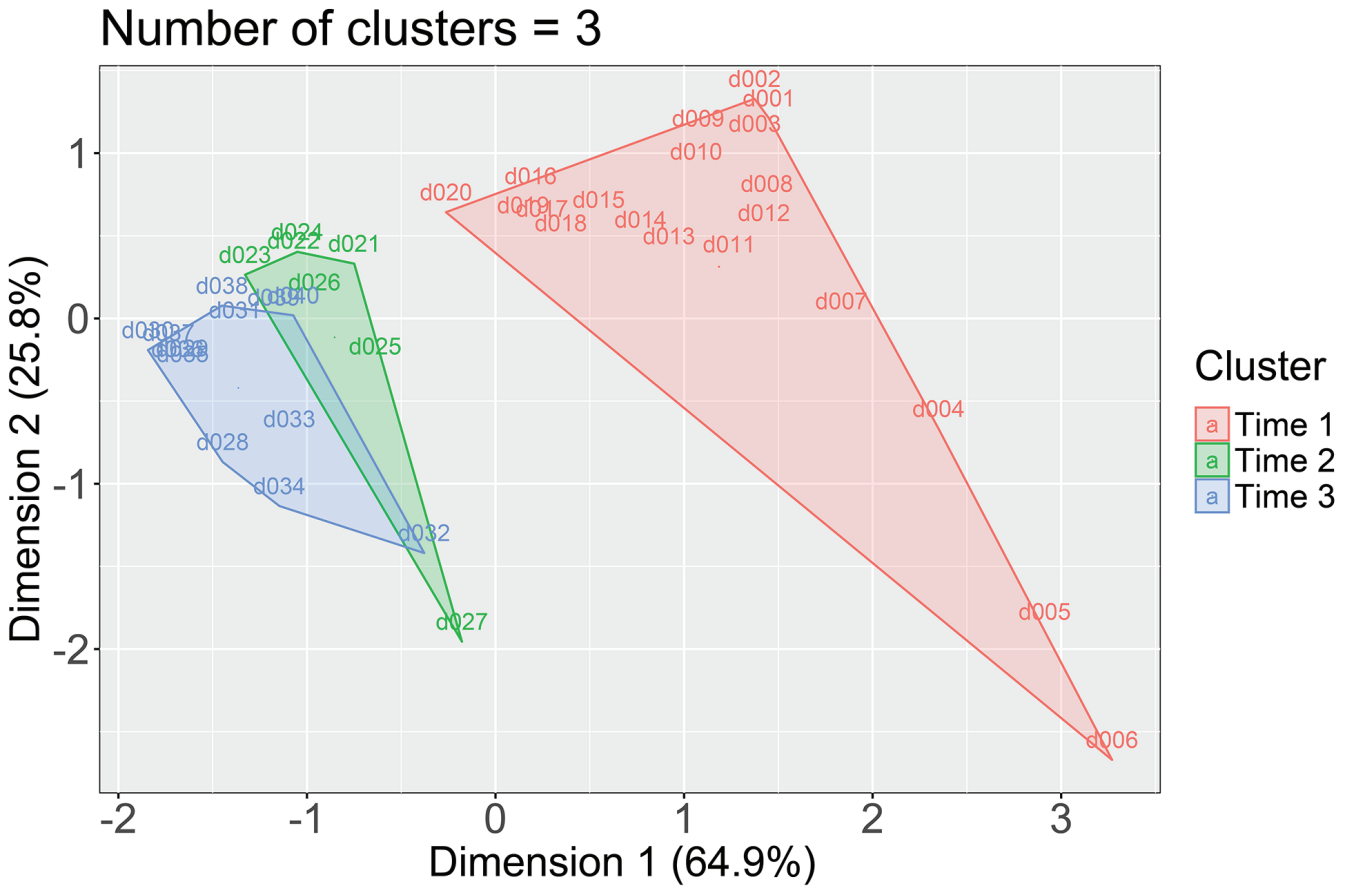
Plot of the three clusters (Time 1, Time 2, and Time 3). Dimension 1 explained 64.9% of the variance; dimension 2 explained 25.8% of the variance.

We used three different methods to determine the number of clusters, namely the elbow method (Bholowalia and Kumar, 2014), the average silhouette method (Rousseeuw, 1987), and the gap statistic method (Tibshirani et al., 2001). The elbow and the gap statistic methods indicated that the best-fitting solution had three clusters, while the average silhouette method indicated that it had two clusters. Given that the two-cluster solution explained 85.3% of the variance and the three-cluster solution explained 95.1% of the variance, we chose the three-cluster solution. The first cluster, named Time 1, grouped the 20 days from 28th February to 18th March; the second cluster, named Time 2, grouped the 7 days from 19th March to 25th March; and the third cluster, named Time 3, grouped the 13 days from 26th March to 7th April. The three clusters are represented in Figure 1.

##### Generalized Linear Models on Clusters

**Figure 2:**
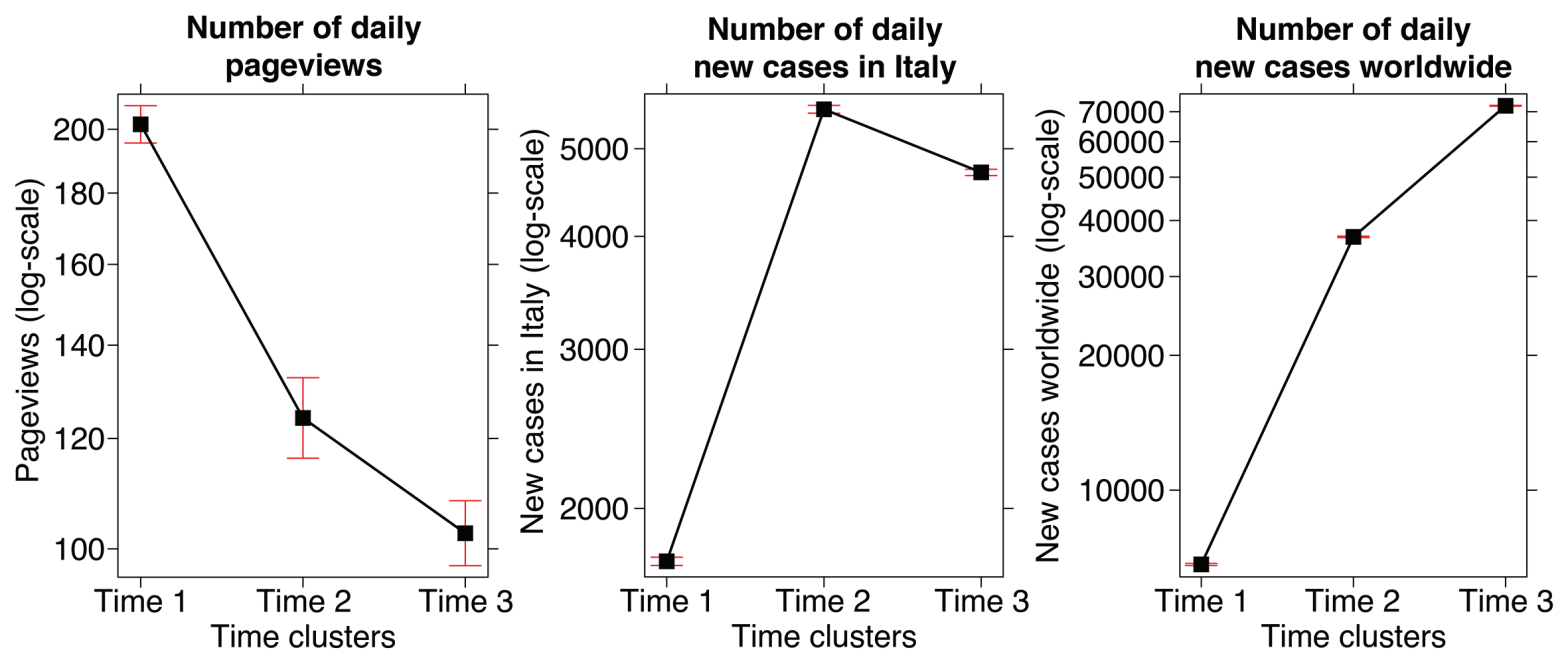
Representation of the three clusters (Time 1, Time 2, and Time 3) concerning the number of daily pageviews of the page of the pamphlet in the HEMOT^®^ website, the number of daily new cases in Italy, and the number of daily new cases worldwide, according to the results of the *k*-means cluster analysis. For each graph, the *y*-axis is represented on a logarithmic scale, and the bars are the 95% confidence intervals.

In order to describe how the three clusters were characterized in terms of the number of daily pageviews and the number of daily new cases, we conducted three GLMs separately for each measure (Figure 2). In each model, we included clusters (Time 1, Time 2, and Time 3) as the between-factor fixed effect and each measure (number of daily pageviews, number of daily new cases in Italy, and number of daily new cases worldwide) as count dependent variables. We utilized the Poisson family and the log link-function. We used Bonferroni correction for *post hoc* tests, and we calculated effect sizes in terms of Cohen’s *d*. The level of significance was *p* < 0.05.

The GLM on the number of daily pageviews revealed a significant effect of clusters, *χ* ^2^(2, *N* = 40) = 563.82, *p* < 0.001. *Post hoc* tests indicated that pageviews were higher, *z* = 12.97, *p* < 0.001, *d* = 0.48, for Time 1 (*M* = 201.65, *SD* = 213.21) compared to Time 2 (*M* = 124.14, *SD* = 153.95), and they were higher, *z* = 4.37, *p* < 0.001, *d* = 0.19, for Time 2 compared to Time 3 (*M* = 102.62, *SD* = 97.83).

Also the GLM on the number of daily new cases in Italy yielded a significant effect of clusters, *χ* ^2^(2, *N* = 40) = 33,215.00, *p* < 0.001. *Post hoc* tests indicated that new cases were lower, *z* = −156.06, *p* < 0.001, *d* = 1.15, for Time 1 (*M* = 1,747.90, *SD* = 1,323.06) compared to Time 2 (*M* = 5,528.57, *SD* = 601.98), while they were higher, *z* = 24.75, *p* < 0.001, *d* = 0.16, for Time 2 compared to Time 3 (*M* = 4,707.69, *SD* = 947.33). However, new cases for Time 3 were higher, *z* = 147.79, *p* < 0.001, *d* = 0.99, than new cases for Time 1.

Finally, clusters resulted significant also in the GLM on the number of daily new cases worldwide, *χ* ^2^(2, *N* = 40) = 1,069,313.00, *p* < 0.001. *Post hoc* tests indicated that new cases were lower, *z* = −502.98, *p* < 0.001, *d* = 1.68, for Time 1 (*M* = 6,825.00, *SD* = 5,571.82) compared to Time 2 (*M* = 36,771.43, *SD* = 8,239.89), and lower, *z* = −302.99, *p* < 0.001, *d* = 0.67, for Time 2 compared to Time 3 (*M* = 72,161.54, *SD* = 7,704.17).

To sum up, during the first part of the campaign (Time 1), the daily pageviews were high while the numbers of daily new cases, both in Italy and worldwide, were quite low; during the central part of the campaign (Time 2), the daily pageviews decreased while the numbers of daily new cases increased; during the last part of the campaign (Time 3), the daily pageviews further decreased, along with a slight diminishment of the daily new cases in Italy and an increase worldwide.

### Perceived Comprehensibility and Utility of the Pamphlet

Concerning comprehensibility, 30.56% of the participants evaluated the pamphlet as very clear, 52.08% as clear, and 17.36% as moderately clear. Moreover, 22.22% of them evaluated it as very useful, 48.61% as useful, 24.31% as moderately useful, and 4.86% as a little useful.

## Discussion and Conclusions

### Discussion of Main Findings

We addressed a public health problem of extreme relevance worldwide in 2020 – the pandemic for which a vaccine or anti-viral treatment had not yet been found at the time in which we conducted the public communication campaign described in this paper. The total traumatic impact of the COVID-19 in terms of loss of human lives, economic changes, and consequences for mental health were still undetermined and potentially very severe. A previously inconceivable uncertainty about the future had suddenly emerged. At both the individual and social levels, negative emotional reactions pervaded communities around the world. This can be stated on the basis of personal experience and anecdotal knowledge, given that scarce data were available to document it at the time of writing. But lessons could be learned from previous research on the consequences of other traumatic events and on the ways used by people to cope successfully with them, specifically findings about epidemics and pandemics different from this one (Cheng and Tang, 2004; Ko et al., 2006; Goodwin et al., 2011; Main et al., 2011; Prati et al., 2011; Vaughan, 2011; Karademas et al., 2012; Manabe et al., 2012; Kim and Niederdeppe, 2013; Taha et al., 2014). Meanwhile, an increasing number of studies on COVID-19 are still in progress (Ahorsu et al., 2020; Galea et al., 2020; Garfin et al., 2020; Horesh and Brown, 2020; Kwok et al., 2020; Shigemura et al., 2020; Wang et al., 2020; Xiang et al., 2020), mirroring the need for urgent research on this topic (Bouffet et al., 2020; Galea et al., 2020; Horesh and Brown, 2020; Xiang et al., 2020).

We were able to plan and implement the campaign described in this article with such a tight schedule, thanks to previous research that we have been conducting to foster disaster-related emotional preparedness among children and adolescents (Raccanello et al., 2019, 2020b,c). Mapping the diffusion of the pamphlet through the media indicated that the campaign was capable of reaching the target population both at a national level and at an international level, through at least 216 media channels including the HEMOT^®^ website. The use of Google Analytics™ data related to the HEMOT^®^ website enabled us to examine characteristics of the visitors of the website and their behavior on the webpage carrying the coronavirus-related pamphlet. More than 6,000 visitors, most from Europe (particularly, Italy) followed by the Americas, visited the website in the first 40 days after the pamphlet publication. The webpage including the pamphlet obtained over 6,200 views, most directly or *via* other websites. The visits were in three waves; most visitors in each wave came from Europe; however, the percentage of visitors from the Americas increased in the second and third waves, mirroring the growing relevance of the problem for those continents. Moreover, the exam of the correlations between the number of daily views of the webpage with the pamphlet and the number of daily new cases in Italy and worldwide suggested the existence of different trends concerning the three measures. Across time, the number of pageviews decreased while the number of new cases in Italy increased, mirroring what was happening worldwide. It is worth noting that the campaign began spreading the pamphlet through the HEMOT^®^ website; however, the following dissemination was prompted also by a variety of other media, independently from the original source. In addition, a cluster analysis enabled us to identify whether the trends relating to pageviews and new cases could be grouped according to specific characteristics. Our findings revealed that the first 40 days of the campaign could be divided into three phases, long 20, 7, and 13 days, respectively. Across the three clusters, the number of daily pageviews decreased; the number of daily new cases in Italy increased from Time 1 to Time 2 while it decreased from Time 2 to Time 3; and the number of daily new cases worldwide continued to increase. We could speculate that the general decrease in pageviews from Time 1 to Time 3, despite the increases in new cases, could be due to a variety of reasons. It does not necessarily mean that the campaign lost its capabilities of reaching people or that people were so overwhelmed that they stopped searching in the Internet for psychological resources. It could be due for example to the overload of daily information spread worldwide related to COVID-19. Alternatively, it could be linked to the fact that many sources independent from our website disseminated the pamphlet, and we had not the possibility to trace them. In the future, it could be useful to verify if this trend is typical of other psychological campaigns of public interest or whether the main source of dissemination remains the most consulted source also in the long-term. Finally, data gathered with a convenience sample of adults who had consulted the pamphlet gave evidence of the clarity of the message conveyed and of the utility of the pamphlet for children and adolescents.

Therefore, these data on the evaluation of the campaign supported its utility. Given the potentially dramatic open-ended implications of the COVID-19 diffusion, future implementation and dissemination of this campaign could be replicated in the same way in different communities and settings. Also, it could be replicated for different public health problems. The experience with this campaign underlines the importance of developing techniques based on demonstrated scientific principles and translating them into forms that can be rapidly deployed in the event of an emergency.

### Limitations and Future Directions

This campaign suffers from several limitations. First, we know that interventions on emotional competence should be tested against the standards of evidence-based research (Flay et al., 2005; Gottfredson et al., 2018) and that they need time and more elaborated activities to increase the probability of changing people’s knowledge, attitudes, and behaviors. Future interventions could also be focused not only on emotions and coping strategies but also on related cognitions and behaviors. Moreover, we did not gather data for assessing the direct impact of the campaign on people’s knowledge and behaviors. However, the much reduced time for releasing the pamphlet to respond promptly to a sudden need due to the unexpected emergency prevented us from respecting all the principles suggested by a scientifically driven persuasion model within health communication. In addition, future materials to be disseminated and interventions should be differentiated, whether possible, according to the specific characteristics of the final addressees, such as age (e.g., for children, adolescents, etc.). In our case, this was not possible, given the urgency of the situation. However, in order to take into account such differences, the pamphlet targeted parents, teachers, etc., as adults who can act as privileged mediators (Masten and Osofsky, 2010) between specific information (given through the pamphlet) and final users. Finally, we highlight that the pamphlet, by being released early in the pandemic, might have received more attention than it would have later, if there were competing sources of information on the same topic, and that is was not possible to monitor in details its dissemination. On the whole, we realize that circulating information is only part of the challenge.

### Conclusion

Finding out how people respond to the information is vitally important, and this should be examined carefully in further studies. Future campaigns could also learn lessons from our experience in this case community study and include these elements to increase their success.

## Data Availability Statement

The raw data supporting the conclusions of this article will be made available by the authors, without undue reservation.

## Ethics Statement

The participants provided their written informed consent to participate in this study.

## Author Contributions

DR, GV, ER, VB, RH, and RB contributed to conception and design of the study and of the pamphlet. DR, GV, and RB organized the database. GV, ER, VB, RH, and RB wrote sections of the manuscript DR wrote the first draft of the manuscript. All authors contributed to manuscript revision, read, and approved the submitted version.

### Conflict of Interest

RH was employed by the company Environmetrics Pty Ltd.

The remaining authors declare that the research was conducted in the absence of any commercial or financial relationships that could be construed as a potential conflict of interest.

## Figures and Tables

**Figure:**
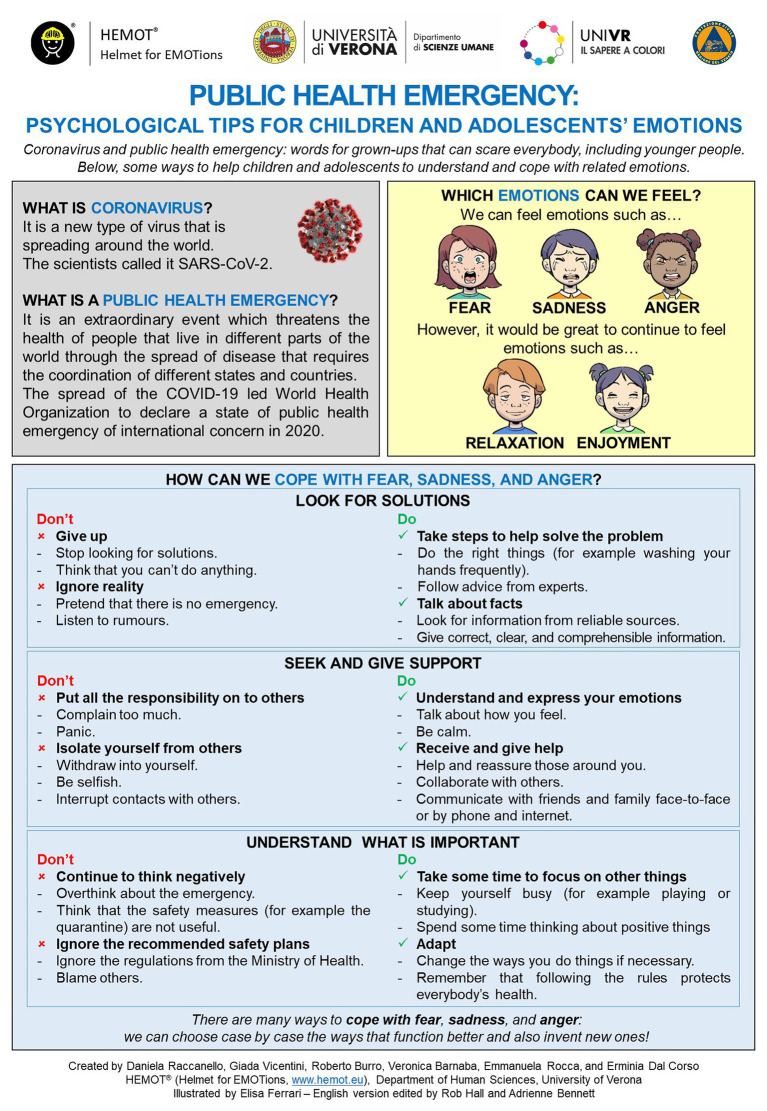


## Footnotes

^1^https://www.hemot.eu/

^2^https://www.hemot.eu/2020/02/28/public-health-emergency/

## Supplementary Material

**SUPPLEMENTARY FIGURE 1** Number of daily pageviews for the webpage dedicated to the pamphlet, number of daily new cases of coronavirus disease (COVID-19) in Italy and worldwide, from 28th February to 7th April 2020.

**SUPPLEMENTARY FIGURE 2** Geographic heat map on the number of pageviews for the webpage dedicated to the pamphlet from 28th February to 7th April 2020 worldwide.
