# Supplementary figures and images for: Development and Early Implementation of a Public Communication Campaign to Help Adults to Support Children and Adolescents to Cope With Coronavirus-Related Emotions: A Community Case Study
Source: Front Psychol. 2020 Sep 10;11:2184. doi: 10.3389/fpsyg.2020.02184 (PMC7511710; doi:10.3389/fpsyg.2020.02184)

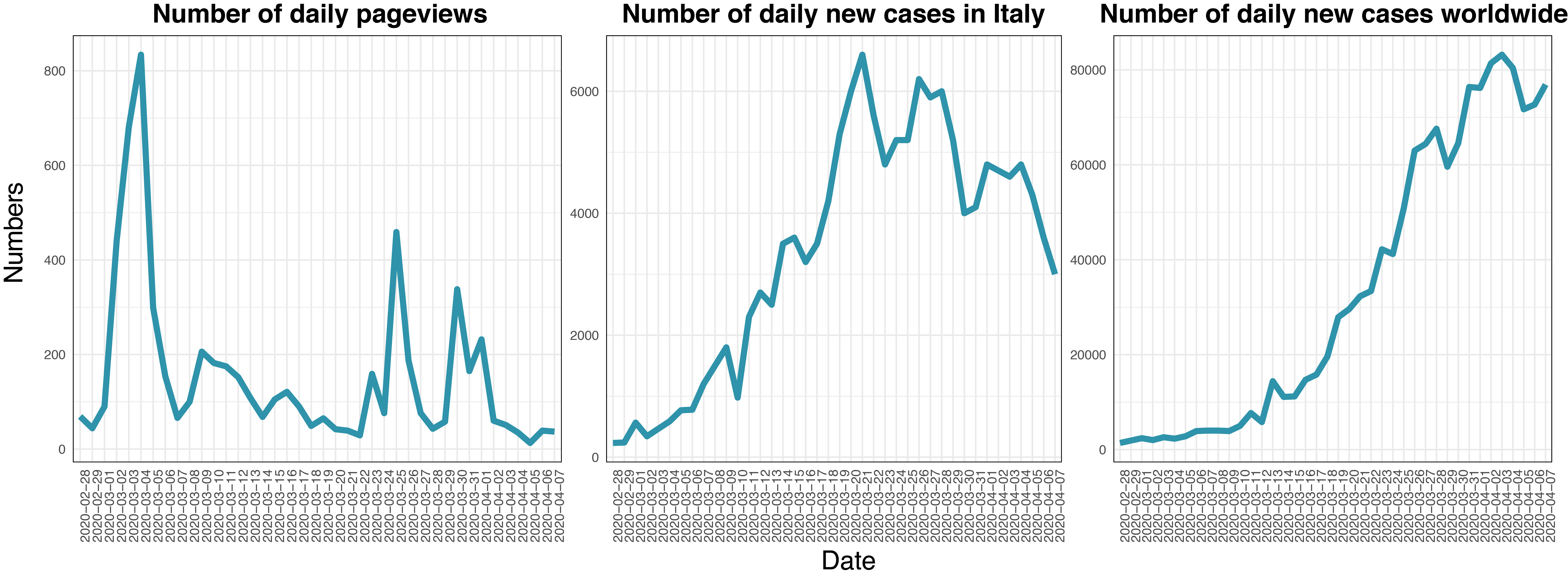

Supplement: Supplementary file 1 [file Image_1.jpeg]

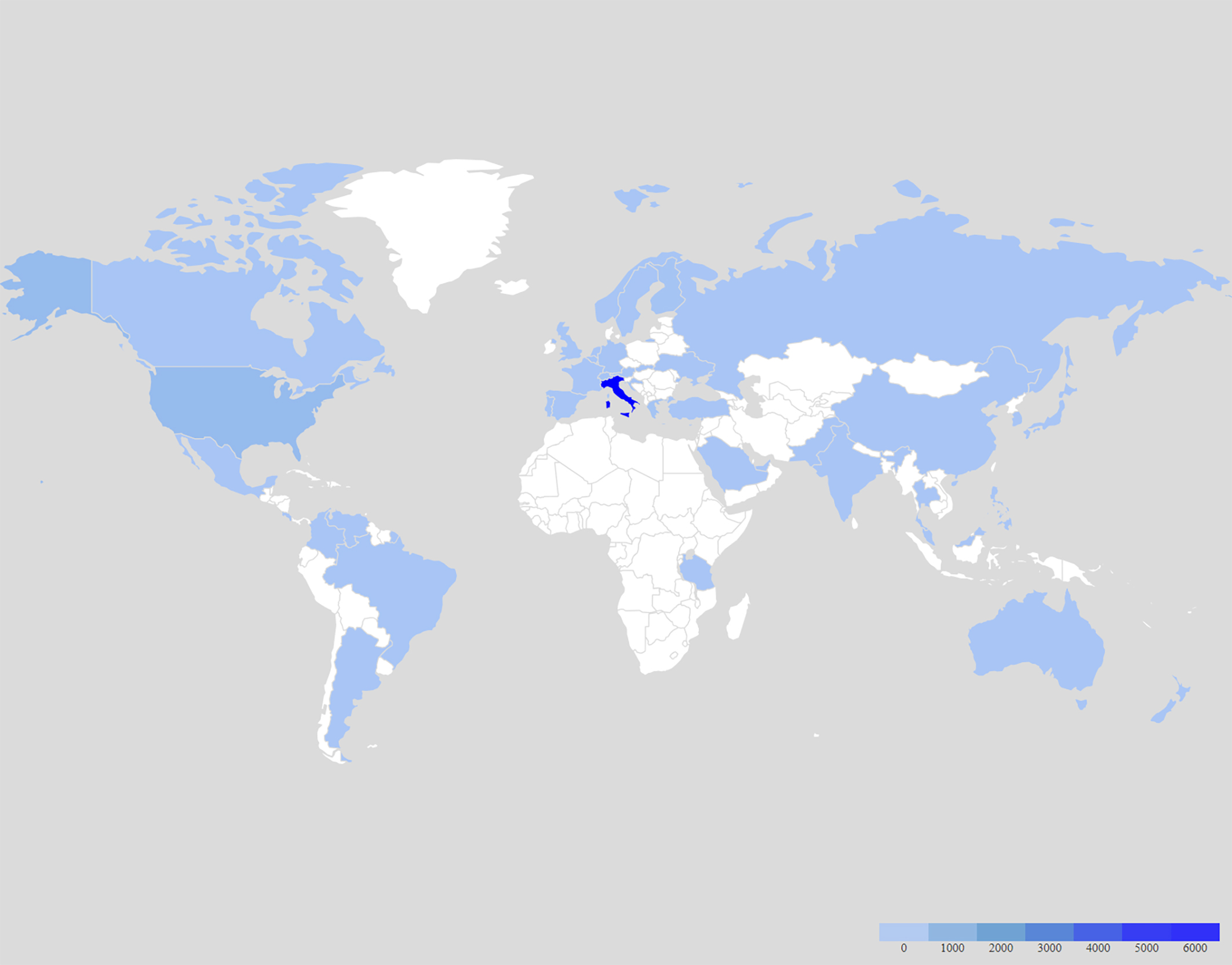

Supplement: Supplementary file 2 [file Image_2.jpeg]
